# Bioinformatics Analysis of miRNAs and mRNAs Network-Xuefu Zhuyu Decoction Exerts Neuroprotection of Traumatic Brain Injury Mice in the Subacute Phase

**DOI:** 10.3389/fphar.2022.772680

**Published:** 2022-06-22

**Authors:** Zhao-yu Yang, Yao Wu, Xuexuan Li, Tao Tang, Yang Wang, Ze-bing Huang, Rong Fan

**Affiliations:** ^1^ Institute of Integrative Medicine, Department of Integrated Traditional Chinese and Western Medicine, Xiangya Hospital, Central South University, Changsha, China; ^2^ National Clinical Research Center for Geriatric Disorders, Xiangya Hospital, Central South University, Changsha, China; ^3^ Department of Infectious Disease, Hunan Key Laboratory of Viral Hepatitis, Xiangya Hospital, Central South University, Changsha, China

**Keywords:** xuefu zhuyu decoction, Bioinformatic analysis, MicroRNAs, traumatic brain injury, neurological recovery

## Abstract

Xuefu Zhuyu decoction (XFZYD) is used to treat traumatic brain injury (TBI). XFZYD-based therapies have achieved good clinical outcomes in TBI. However, the underlying mechanisms of XFZYD in TBI remedy remains unclear. The study aimed to identify critical miRNAs and putative mechanisms associated with XFYZD through comprehensive bioinformatics analysis. We established a controlled cortical impact (CCI) mice model and treated the mice with XFZYD. The high-performance liquid chromatography-tandem mass spectrometry (HPLC-MS/MS) confirmed the quality of XFZYD. The modified neurological severity score (mNSS) and Morris water maze (MWM) tests indicated that XFZYD improved the neurological deficit (*p* < 0.05) and cognitive function (*p* < 0.01). Histological analysis validated the establishment of the CCI model and the treatment effect of XFZYD. HE staining displayed that the pathological degree in the XFZYD-treated group was prominently reduced. The transcriptomic data was generated using microRNA sequencing (miRNA-seq) of the hippocampus. According to cluster analysis, the TBI group clustered together was distinct from the XFZYD group. Sixteen differentially expressed (5 upregulated; 11 downregulated) miRNAs were detected between TBI and XFZYD. The reliability of the sequencing data was confirmed by qRT-PCR. Three miRNAs (mmu-miR-142a-5p, mmu-miR-183-5p, mmu-miR-96-5p) were distinctively expressed in the XFZYD compared with the TBI and consisted of the sequencing results. Bioinformatics analysis suggested that the MAPK signaling pathway contributes to TBI pathophysiology and XFZYD treatment. Subsequently, the functions of miR-96-5p, miR-183-5p, and miR-142a-5p were validated *in vitro*. TBI significantly induces the down-expression of miR-96-5p, and up-expression of inflammatory cytokines, which were all inhibited by miR-96-5p mimics. The present research provides an adequate fundament for further knowing the pathologic and prognostic process of TBI and supplies deep insights into the therapeutic effects of XFZYD.

## GRAPHICAL ABSTRACT

A multi-stage analysis methodology was employed in the present research. (TBI: traumatic brain injury; XFZYD: Xuefu Zhuyu Decoction; CCI: controlled cortical impact; DEMs: differential expressed miRNAs; GO: Gene Ontology; KEGG: Kyoto Encyclopedia of Genes and Genomes).
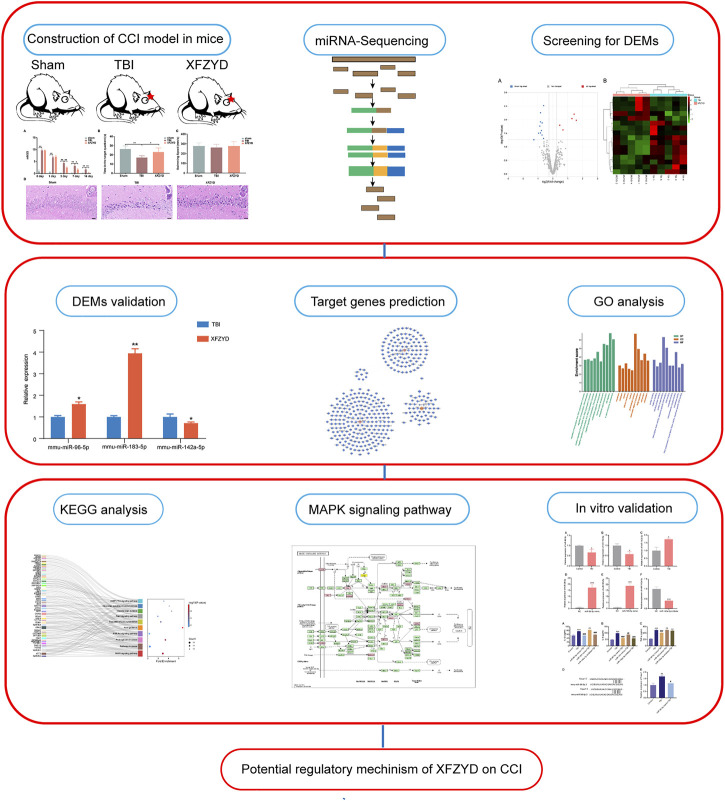

## Introduction

Traumatic brain injury (TBI) remains a major cause of morbidity and mortality worldwide (Khellaf et al., 2019). Based on a survey, the population of TBI patients in China will exceed 1.39 billion, accounting for approximately 18% of the world population (Jiang et al., 2019). Higher rates of death and disability make TBI a global health challenge (Khellaf et al., 2019). The pathogenesis of TBI is a complex process including primary and secondary injuries, which make it incredibly challenging to treat (Jassam et al., 2017). Multitudinous studies have been conducted to search for the therapy of TBI. Disappointingly, these preclinical experimental researches have not been effectively converted to clinical treatment (Galgano et al., 2017) partly because of the comprehensive influence of various complicated secondary biochemical and pathophysiological cascade reactions happening at different times points (Lagraoui et al., 2017; Wang et al., 2018). Therefore, understanding complex pathophysiological and exploring optimal therapies after TBI has become meaningful.

Among the critical pathophysiological processes of TBI are the learning and memory deficits due to the injury (Wu et al., 2013). The hippocampus plays a crucial role in learning and memory while being extremely susceptible to TBI (Weston et al., 2021). Specifically, hippocampal volume reduction has been observed in TBI patients (Bae et al., 2020). The hippocampus is frequently discussed in brain subfields in TBI because of its vital parts in short-term and spatial-related memories (Shokouhi et al., 2020). Although multiple strategies have been explored to improve cognitive impairment after TBI (Yu et al., 2008; Patel and Sun, 2016), the current therapies are far from satisfactory (Konrad et al., 2011). It is necessary to consider the different natural therapeutic methods. Xuefu Zhuyu Decoction (XFZYD), a classical prescription, has been widely used in clinical to treat cardiac-cerebral vascular disease (Zhang et al., 2004; Yang T. et al., 2019; Fu et al., 2020; Wang et al., 2020a). Our previous studies reported that XFZYD could reduce neurological deficits after TBI via inflammatory inhibition (Xing et al., 2016) and improve the long-term prognosis post-TBI via synaptic regulation. (Zhu et al., 2018). Several scientists have explored the potential therapeutic effects of XFZYD, which may alter the protein and metabolites expression (Li et al., 2020; Zheng et al., 2020) in the hippocampus after TBI. Nevertheless, there might be a disconnection between the mRNAs’ expression and their resultant proteins (Gygi et al., 1999; Redell et al., 2009). The variations in mRNA and protein expression levels may be attributed to non-coding RNAs (ncRNAs) affection (Redell et al., 2009).

MicroRNAs (miRNAs), a class of small ncRNAs, govern a variety of physiological and pathological processes such as development, differentiation, metabolism, and apoptosis (Huang et al., 2011). miRNAs are essential ncRNAs abundant in the brain to regulate genes transcription and associated molecules expression (Yang Y. et al., 2019; Rahmati et al., 2021). What’s more, microRNAs (miRNAs), are a well-known diagnostic tool both in the clinical setting and in the medico-legal investigation (Sessa et al., 2019). Notably, investigations demonstrated that miRNAs levels are altered in the acute phase of TBI (Raghavendra Rao et al., 2003; Redell et al., 2009; Xiao et al., 2020). Previous medical investigations indicated that miRNAs may act as possible targets for disease progress evaluation and interference against TBI to alleviate impairment to the cerebrum (Martinez and Peplow, 2017). Integrated bioinformatics analysis has identified several molecules and pathways in rats’ hippocampus after TBI during the acute stage (Xiao et al., 2020). Nonetheless, no research discusses the miRNAs’ alteration in the hippocampus during the subacute phase of TBI. Unlike other diseases, TBI consists of a time-dependent range of events. The diverse alterations in the impaired area, including vascular injury, microglial polarization, neuronal death, and astrocyte activation, have been demonstrated to vary over time with different molecular expression modes (Algattas and Huang, 2013; Jassam et al., 2017). Thus, investigating miRNAs expression patterns in the subacute phase facilitates our knowledge of the underlying molecular mechanisms and the potential treatment after TBI.

In the current research, we explored the expression patterns of miRNAs in TBI and XFZYD-treated groups. First, the miRNAs-sequencing was applied to test the differential expression spectrum of miRNAs between the controlled cortical cortex (CCI) model and the animals treated with XFZYD. Next, we used bioinformatics analysis to investigate several differentially expressed miRNAs’ biological activities to uncover possible treatment pathways for XFZYD. The current study will provide unique insights into seeking the essential mechanisms in the XFZYD treating TBI.

## Materials and Methods

### Xuefu Zhuyu Decoction Preparation

XFZYD was purchased from Xiangya Hospital Central South University (batch number: 20,190,415, Hunan Zhenxing Traditional Chinese Medicine Co., Ltd.). Professor Suiyu Hu (the Institute of Integrative Medicine of Xiangya Hospital Central South University), a herbal medicinal botanist, authenticated each herb of XFYZD (Xing et al., 2016; Li et al., 2020). XFZYD comprises eleven crude drugs: Prunus persica (L.) Batsch (Tao Ren), Carthamus tinctorius L. (Hong Hua), Angelicae sinensis (Oliv.) Diels (Dang Gui), Rehmannia glutinosa Libosch. (Sheng Di), *Achyranthes* bidentata Bl. (Niu Xi), Paeonia lactiflora Pall. (Chi Shao), Citrus aurantium L. (Zhi Qiao), *Glycyrrhiza* uralensis Fisch. (Gan Cao), Ligusticumi chuanxiong Hort. (Chuan Xiong), Platycodon grandiflorum (Jacq.) A. DC. (Jie Geng), and Bupleurum chinense DC.(Chai Hu). The detailed information of drugs was recorded in Table 1. The soaking of herbs was performed in a six-times volume of ddH_2_O (*w/v*) for 0.5 h and subsequently boiled twice, followed by combining the two boiled solutions. The final concentration was 0.75 g/ml for intragastric administration.

**TABLE 1:** Composition of xuefu zhuyu decoction (XFZYD).

Botanical Name	Chinese Name	Medical Part	Ratio	Specimen Number
Prunus persica (L.) Batsch	Tao Ren	Seed	8	19,061,010
Carthamus tinctorius L.	Hong Hua	Flower	6	19,080,108
Angelica sinensis (Oliv.) Diels	Dang Gui	Root	6	19,081,303
Rehmannia glutinosa (Gaertn.) DC.	Sheng Di	Root	6	19,051,007
*Achyranthes* bidentata Blume.	Niu Xi	Root	6	19,041,505
Paeonia lactiflora Pall.	Chi Shao	Root	4	19,062,607
Citrus × aurantium L.	Zhi Qiao	Fruit	4	19,051,003
*Glycyrrhiza* uralensis Fisch.	Gan Cao	Root	4	19,080,611
Ligusticum striatum DC.	Chuan Xiong	Root	3	19,062,904
Platycodon grandiflorus (Jacq.) ADC.	Jie Geng	Root	3	19,061,512
Bupleurum chinense DC.	Chai Hu	Root	2	19,052,910

The botanical names have been checked with http://www.theplantlist.org.

### Qualitative Analysis of Xuefu Zhuyu Decoction

We purchased amygdalin, neohesperidin, rutin, and Digoxin from Yuanye Bio-Technology Co., Ltd. (Shanghai, China). Digoxin is a reference compound because it is not the internal composition of XFZYD and plasma; Digoxin does not disturb the residence times of the three objects. HPLC-MS/MS system (Shimadzu 8,050, Kyoto, Japan) was used for qualitative analysis in negative ion mode. After adding acetonitrile, mixing Digaoxin with the plasma samples, vortex the mixture (1 min), and centrifuge the mixture (13,000 rpm, 15 min, 4°C). Using a nitrogen dryer to dry the supernatants, diluted the dried supernatants (10% acetonitrile-water) and injected them into the HPLC-MS/MS for detection.

### Controlled Cortical Impact Model

Whole experimental plans were conducted following the Animal Care Committee of Central South University (Changsha, China) and the National Institutes of Health Guidelines for the Care and Use of Laboratory Animals. The male adult mice (C57BL/6J, weight 25–30 g) used in the present experiment were obtained from the Department of laboratory Animals, Central South University (Changsha, China). Mice were adequately housed in a clean environment with appropriate temperatures and fed with standard rodent food and purified water. According to previous reports, the CCI model was constructed (Treangen et al., 2018) with slight modification. Mice were deeply anesthetized with 0.3% sodium pentobarbital (50 mg/kg, intraperitoneally). Then the 25–30 g mice were subjected to the CCI model using the TBI-03101 (Precision Systems and Instrumentation, Fairfax Station, VA). The impact parameters were 1.0 mm depth, 3.5 m/s speed, and 80 ms dwell time. Finally, closing the incision with sutures. To maintain the mice’s body temperature, all mice after surgery were placed on a warm blanket to keep their temperature at 37.0 ± 0.5°C. The sham group only underwent anesthesia and craniotomy but without brain impact.

### Modified Neurological Severity Score Test

Using the mNSS test evaluated the neurological functional outcomes. Two investigators completed the mNSS test of mice after surgery and on days 1, 3, 7, and 14 after XFZYD treatment. The degree was graded from 0 to 18 (normal score, 0; maximal deficit score, 18).

### Morris Water Maze Test

Assessment of cognitive function was using the MWM test (Vorhees and Williams, 2006), as previously described (Zhang et al., 2017a). The pool was filled with water. The water temperature was kept at approximately 22 ± 2°C. Mice were training four times per day for five consecutive days. The tested mice were positioned facing the tank wall starting from four different locations (north, south, east, west). A computerized video tracking system (ANY-maze, Stoelting Co., United States) was used to record the animal’s swimming speed and time in the target quadrant.

### Hematoxylin and Eosin Staining

We were using xylene dewaxed the brain sections (each 5-μm). Then the gradient ethanol was applied to hydrate the brain sections. Next, staining the brain with HE reagent (Solarbio, Beijing, China). A light microscope was applied to examine the morphology of hippocampal neurons.

### Ribonucleic Acid-Sequencing (Ribonucleic Acid-Seq)

The miRNAs expression profiles were obtained as previously described (Zhang et al., 2019). Briefly, using TRIzol reagent to extract the total RNA from the hippocampus in accordance with the manufacturer’s instructions. NanoDrop 2000 spectrophotometer was applied to quantify the concentration of extracted RNA. The NEB Next Ultra Directional RNA Library Prep Kit for Illumina (NEB, MA, United States) was used to establish an RNA library and assessed the RNA library quality and quantity via Agilent 2,100 Bioanalyzer. The RNA library was used for sequence analysis. The sequence analysis was conducted by a NextSeq 500 platform (Illumina, CA, United States). The clean reads were filtered out from raw reads by FastQC and selected for further bioinformatics analysis. Significant miRNAs were selected with a cutoff of log_2_ (fold change) > 0.3 and *p*-value < 0.05.

### Target Genes Prediction and Bioinformatics Analysis

TargetScan (http://www.targetscan.org/) and miRDB (http://www.mirdb.org/miRDB/), two online analysis tools, were applied to predict the target genes of miRNAs. To further understand the predicted target genes’ function, we applied Gene Ontology (GO) and Kyoto Encyclopedia of Genes and Genomes (KEGG) pathway enrichment analysis by using the Database for Annotation, Visualization, and Integrated Discovery version (DAVID) (https://david.ncifcrf.gov/). The standard cut-off criterion was *P* < 0.05. The miRNAs-genes network was established and displayed via Cytoscape software (version 3.7.2, http://www.cytoscape.org/download.php).

### Quantitative Reverse Transcriptase-Polymerase Chain Reaction

To confirm the reliability of RNA-seq results, we used qRT-PCR to detect the relative expressions of miRNA. Using independent groups of animals, hippocampi were harvested after TBI and XFZYD (*n* = 5, each group). The U6 gene was a reference control. Using the comparative Ct (2^−ΔΔCt^) method (Livak and Schmittgen, 2001) to calculate the relative expression of miRNAs. The sequences of primers are listed in Table 2.

**TABLE 2:** Reverse-transcription polymerase chain reaction primers.

Name	Primers
U6	F: 5′ GCT​TCG​GCA​GCA​CAT​ATA​CTA​AAA​T 3′
	R: 5′ CGC​TTC​ACG​AAT​TTG​CGT​GTC​AT 3′
mmu-miR-142a-5p	F: 5′ GGG​GGG​CAT​AAA​GTA​GAA​AGC 3′
	R: 5′ GTGCGTGTCGTGGAGTCG 3′
mmu-miR-96-5p	F: 5′ GGTTTGGCACTAGCACAT 3′
	R:5′ CAGTGCGTGTCGTGGAGT 3′
mmu-miR-183-5p	F:5′ GGG​GTA​TGG​CAC​TGG​TAG​AA 3′
	R: 5′ GTGCGTGTCGTGGAGTCG 3′
Rasa1	F: 5′ GAA​CTT​GGG​AAT​GTA​CCT​GAA​C 3′
	R: 5′ TGT​GCA​CCA​CGC​TCA​TTA​C 3′

### Cell Culture and Scratch-Injury Model

BV-2 microglial cells (Procell, Wu Han, China) were cultured in Dulbecco’s modified eagle medium supplemented with 10% fetal bovine serum in a humidified incubator under 5% CO_2_ at 37°C. Cells were split at 70–80% confluence before the following experiments.

To study the impact of miR-96-5p, miR-183-5p, and related miR-142a-5p after TBI *in vitro*, a scratch injury model was used as previously reported (Han et al., 2014; Huang et al., 2018). Confluent cultured BV-2 cells were scratched across the cell surface (both vertically and horizontally with a 4-mm space between each line) using a 10 μL pipette tip, and detached cells were removed by washing with PBS.

### Cell Transfection

miR-96-5p mimic, miR-183-5p mimic, and miR-142a-5p inhibitor, as well as their corresponding negative control (NCs), were designed by RiboBio Co., Ltd. (Guangzhou, China). BV-2 microglial cells (1.5×10^5^ cells/ml) in a 6-well plate were transfected with 50 nM miR-96-5p mimic, 50 nM miR-183-5p mimic, 100 nM miR-142a-5p inhibitor or their NCs by using a Lipofectamine 3,000 reagent (Invitrogen, Carlsbad, CA, United States) according to the manufacturer’s instructions. Transfected cells were incubated for an additional 24 h prior to the scratch injury. Corresponding sequences were as follows: miR-96-5p mimic, 5′UUU​GGC​ACU​AGC​ACA​UUU​UUG​CU3’; miR-183-5p mimic, 5′ UAU​GGC​ACU​GGU​AGA​AUU​CAC​U 3’; mimic-NC, 5′ UUU​GUA​CUA​CAC​AAA​AGU​ACU​G3’; miR-142a-5p inhibitor, 5′ GUA​UUU​CAU​CUU​UCG​UGA​UGA 3’; inhibitor-NC, 5′ CAG​UAC​UUU​UGU​GUA​GUA​CAA​A 3’. The efficiency of transfections was validated by comparing the levels of miR-96-5p, miR-183-5p, and miR-142a-5p between transfected and controlled cells by quantitative real-time-polymerase chain reaction (qRT-PCR).

### Enzyme-Linked Immunosorbent Assay

To evaluate the inflammatory response in injured BV-2 cells, the cell culture medium was gathered 24 h after scratch injury. ELISA of inflammatory mediators, including IL-1β, TNF-α, and IL-6 were performed according to the manufacturer’s instructions (Renjie Bio, Shanghai, China).

### Statistics Analysis

All data are expressed as the mean ± SD. Data were analyzed using SPSS 26.0. Statistical analysis was analyzed by one-way variance (ANOVA) followed by Turkey’s *post hoc* tests. For the comparison between two groups, data were analyzed with standard two-tailed unpaired t-tests. *p*-value < 0.05 was considered statistically significant.

## Results

### The Qualitative Analysis of Xuefu Zhuyu Decoction

**FIGURE 1:**
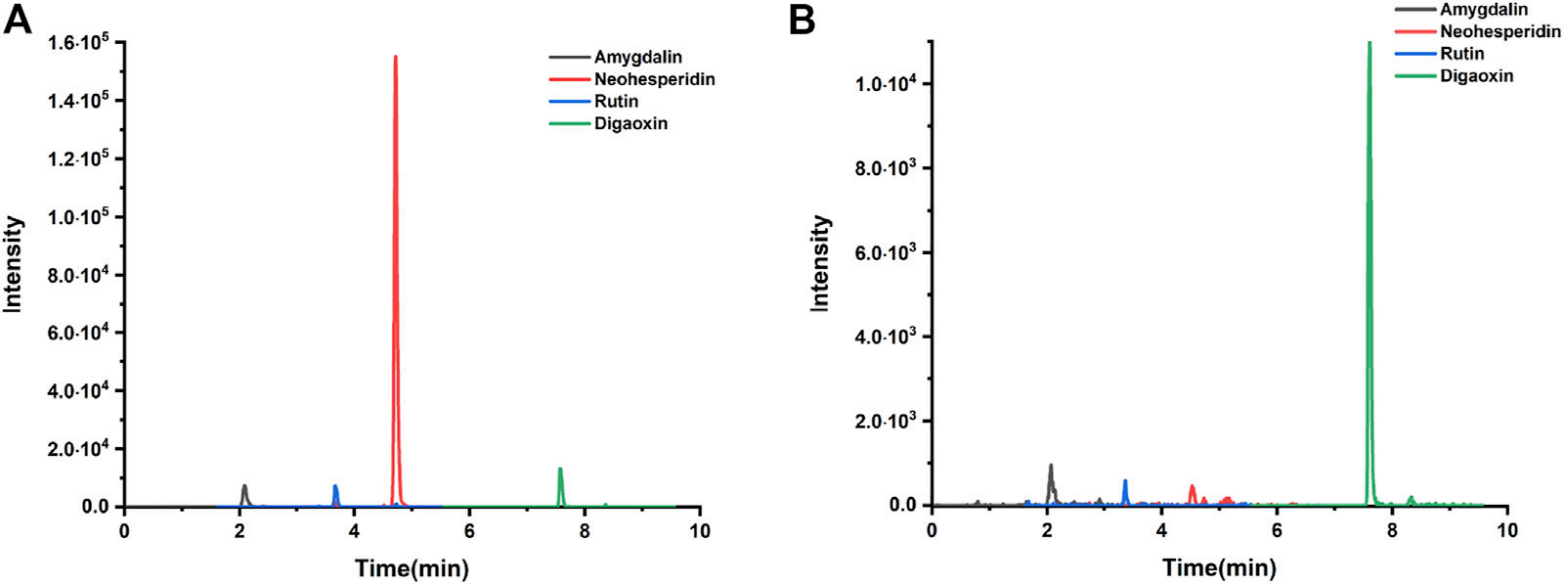
Qualitative analysis. **(A)** Amygdalin, neohesperidin, rutin, and digaoxin of standard agents detected by LC chromatogram. **(B)** Amygdalin, neohesperidin, rutin, and digaoxin of XFZYD in plasma samples post-CCI.

HPLC-MS/MS was used to investigate the herbal quality of XFZYD. Amygdalin, rutin, neohesperidin, and Digoxin (internal reference) were detected (Figures 1A,B). The retention time of amygdalin, rutin, neohesperidin, and Digoxin was 2.09 ± 0.03 min, 3.68 ± 0.02 min, 4.52 ± 0.005 min, and 7.64 ± 0.002 min, respectively (Figure 1B). The coefficients of variation of the four ingredients were less than 2%, indicating the stability of the method.

### Xuefu Zhuyu Decoction Improves Neurological Recovery and Alleviated Cognitive Dysfunction

**FIGURE 2:**
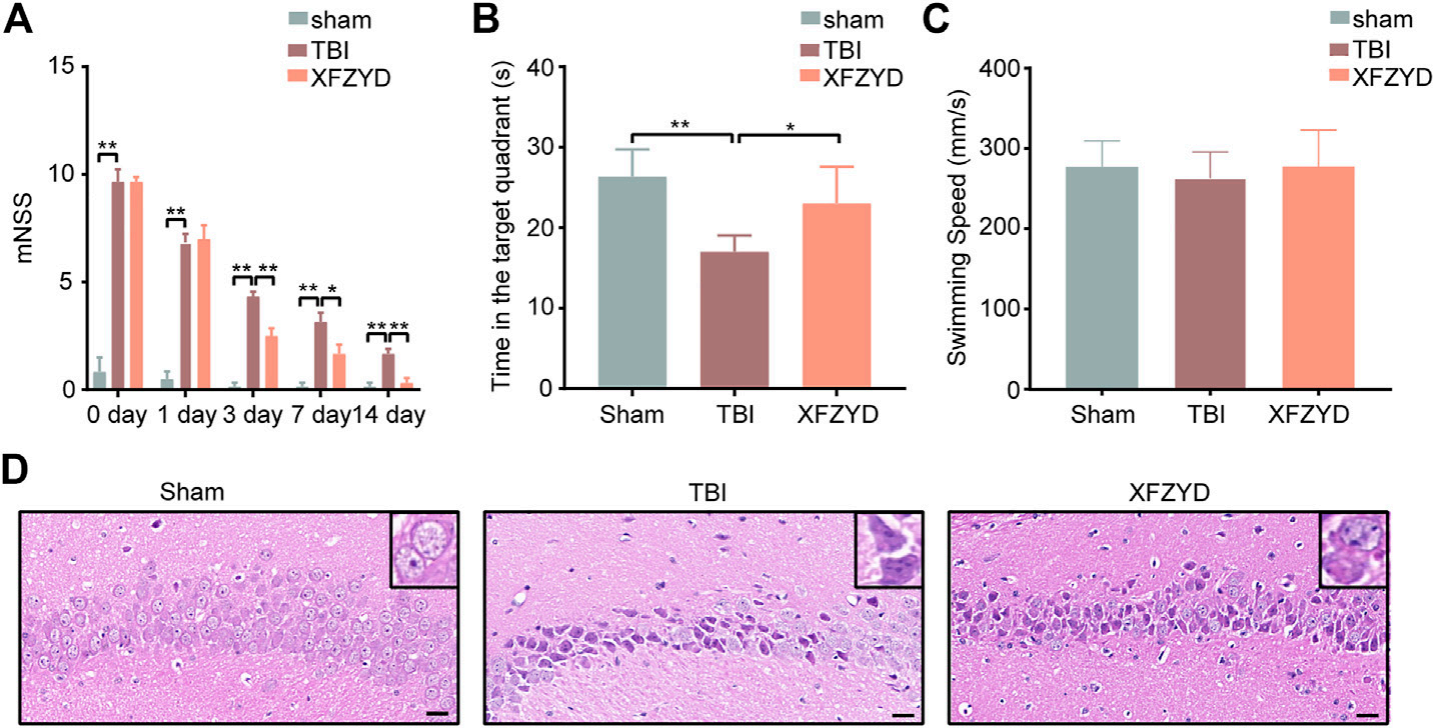
Effects of XFZYD on TBI. **(A)** The mNSS of sham, TBI, and XFZYD groups on post-injury, 1st day, 3rd day, 7th day, and 14th day. (0 day presents post-injury) **(B)** Time in the target quadrant of sham, TBI, and XFZYD groups. **(C)** The swimming speed of sham, TBI, and XFZYD groups. **(D)** HE staining (400 x) in CA1 region on 14th day, scar bar = 20 μm. Data are displayed as mean ± SD, *n* = 6 mice per group, *p < 0.05, **p < 0.01.

To evaluate the effects of XFZYD in TBI mice’s neural functional recovery, we adopted the mNSS test, including five parts (motor, sensor, reflex, and equilibrium sense) to assess neurological deficits. The mNSS score of sham, TBI, and XFZYD groups was summarized in Figure 2A. The mice subjected to CCI showed similar neurological deficiencies on the 1st day. The score of the XFZYD group decreased relative to that of the TBI group on the 3rd (*p* < 0.01), 7th (*p* < 0.05), and 14th days (*p* < 0.01) (Figure 2A). The results indicate that XFZYD could promote neural functional recovery.

The MWM test was used to assess hippocampus-dependent reference learning and memory ability (Chohan et al., 2015). The TBI mice exhibited significantly less dwell time in the target quadrant than a sham and XFZYD groups, indicating the retention of spatial and acquired memory was impaired (Figure 2B). A single-factor ANOVA revealed none of the groups’ swimming speeds significantly differed (Figure 2C). The result suggests differences of time in the target quadrant were not due to injury-induced motor impairment.

The morphology of the hippocampus (CA1 region) was observed by HE staining. The outcomes state that the morphology of the sham group was the round and intact nucleus. In the TBI group, there was severe nuclear concentration, loose staining, and cell death. The pathological degree of the CA1 region in the XFZYD-therapy group was prominently reduced contrasted the TBI group (Figure 2D).

### Xuefu Zhuyu Decoction Altered the miRNAs Expression Profiles in the Hippocampus of Traumatic Brain Injury Mice

**FIGURE 3:**
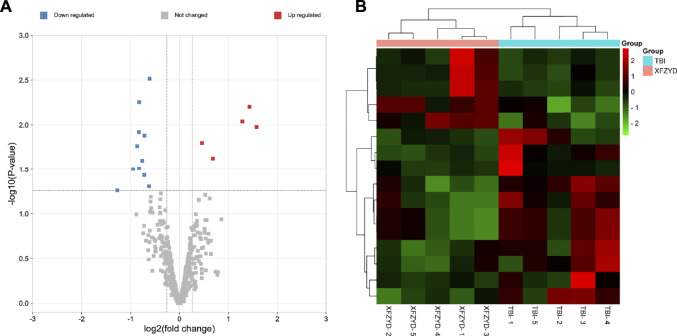
**(A)** Volcano plot of distinctively expressed miRNA in XFZYD/TBI. The red dots represent upregulated miRNAs, and the blue dots represent downregulated miRNAs (log2 (fold change) > 0.3 and *p*-value < 0.05). **(B)** Hierarchical clustering of DEMs in XFZYD/TBI. Ten samples were obtained from the TBI group and XFZYD group. Hierarchical clustering was formed based on miRNAs’ expression levels. Green, downregulated; red, upregulated (*n* = 5 mice per group). TBI: traumatic brain injury; XFZYD: Xuefu Zhuyu decoction.

To understand the underly mechanism of XFZYD treatment, miRNA sequencing was applied to establish miRNA expression profiles of TBI and XFZYD groups. The RNA-seq data have been deposited into GEO (GSE198915). With the threshold of log_2_ (fold change) > 0.3 and *p*_value <0.05, 16 differentially expressed miRNAs were detected. Among the distinctively changed miRNAs, five were upregulated, and 11 were downregulated in the XFZYD-treated class (Figure 3A and Table 3). To further examine these differentially expressed miRNAs, we constructed a hierarchical clustering map. The five TBI groups clustered together in one group were primarily distinct from the XFZYD groups. Overall, changes in the state from the TBI group to the XFZYD group were also separated by differences in expression profiles of miRNA (Figure 3B).

**TABLE 3:** Differentially expressed miRNAs between TBI and XFZYD-treated mice in hippocampus tissue.

Mature_ID	Fold_Change	*p*_value	Regulation
mmu-miR-96-5p	2.978,383,826	0.01,055,772	up
mmu-miR-182-5p	2.695,817,823	0.006269	up
mmu-miR-183-5p	2.435,777,511	0.00913,512	up
mmu-miR-7015-3p	1.606,408,239	0.02,381,847	up
mmu-miR-296-3p	1.377,959,652	0.0159,655	up
mmu-miR-383-5p	0.652,210,584	0.00304,676	down
mmu-miR-142a-5p	0.648,070,417	0.04,930,959	down
mmu-miR-33-5p	0.607,919,971	0.01,316,971	down
mmu-miR-466d-5p	0.605,773,732	0.03,672,384	down
mmu-miR-466n-5p	0.605,773,732	0.03,672,384	down
mmu-miR-3083b-3p	0.58,879,273	0.02,518,238	down
mmu-miR-1251-5p	0.564,439,115	0.00556,832	down
mmu-miR-551b-5p	0.562,474,965	0.03,081,071	down
mmu-miR-200b-3p	0.561,310,787	0.01,206,024	down
mmu-miR-376a-3p	0.547,921,578	0.01,728,533	down
mmu-miR-429-3p	0.517,731,483	0.03,141,041	down

### Validation of Candidate miRNAs by Quantitative Reverse Transcriptase-Polymerase Chain Reaction

**FIGURE 4:**
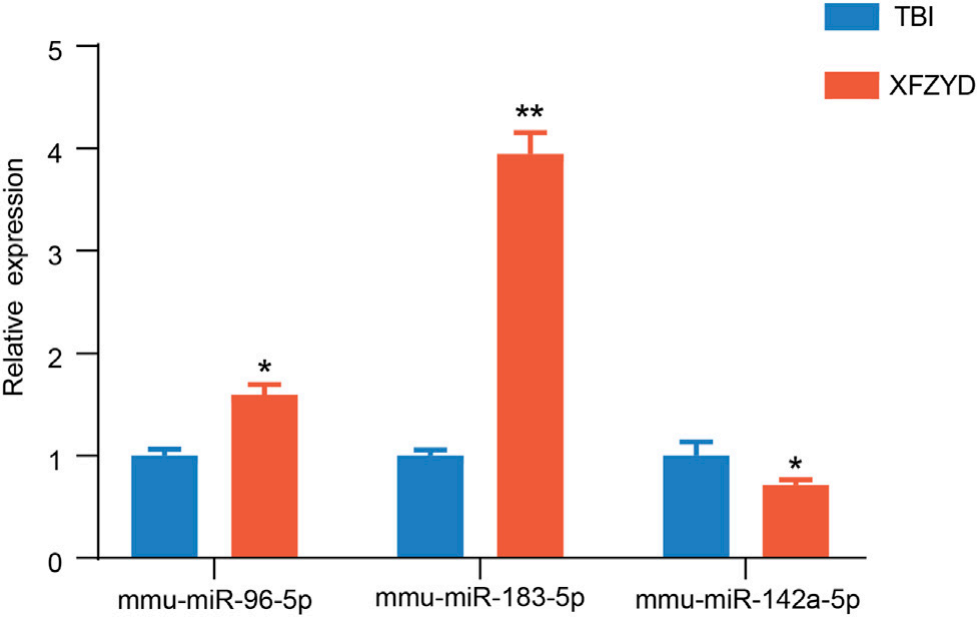
Validation of the RNA-sequencing data. Statistical difference analysis of the relative expression levels of miR-96-5p, miR-183-5p, and miR-142a-5p in TBI and XFZYD groups. Data are exhibited as mean ± SEM, n = 5 mice per group, *p < 0.05, **p < 0.01.

To confirm the result of the sequencing, three miRNAs were examined by qRT-PCR. One downregulated miRNA (mmu-miR-142a-5p), and two upregulated miRNAs (mmu-miR-183-5p, mmu-miR-96-5p) were chosen for qRT-PCR (Figure 4). The results of qRT-PCR were consistent with our sequencing results. Three miRNAs were distinguishedly expressed in the XFZYD group relative to the TBI group (mmu-miR-142a-5p, *p* < 0.05; mmu-miR-183-5p, *p* < 0.01; mmu-miR-96-5p, *p* < 0.05), indicating the reliability of the sequencing data.

### Target Gene Prediction and Integrated Network Analysis

**FIGURE 5:**
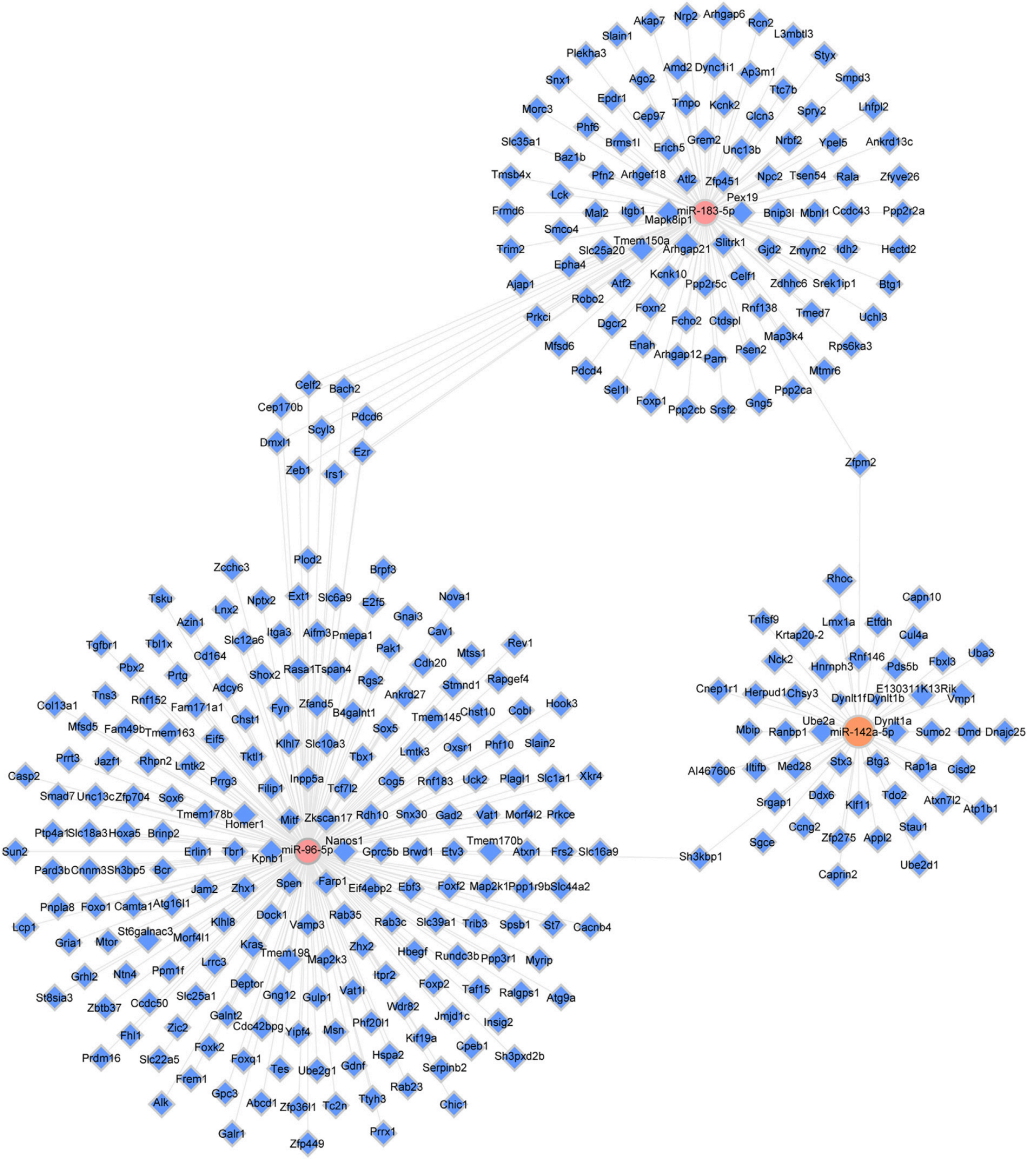
The miRNA-target genes network. Pink dots represent upregulated miRNAs (miR-96-5p and miR-183-5p); orange nodes represent downregulated miRNAs (miR-142a-5p); blue dots represent target genes, and solid gray lines illustrate correlations between miRNAs and target genes.

One miRNA had enough target multiple genes, while a single gene, in turn, was able to associate with various miRNAs. TargetScan and miRDB were applied to acquire the target genes. The intersection of the two online tools was considered as the final result. In the present study, to acquire further insight into the underlying therapeutic mechanism of XFZYD, we chose the three validated miRNAs (mmu-miR-142a-5p, mmu-miR-183-5p, and mmu-miR-96-5p) to construct the miRNAs-target genes network. (Figure 5). The upregulated miRNA mmu-miR-183-5p was related to 105 mRNAs, and mmu-miR-96-5p was associated with 206 mRNAs. Meanwhile, the downregulated miRNAs, including mmu-miR-142a-5p were linked to 49 mRNAs (Figure 5).

### Functional Examination of Target Genes

**FIGURE 6:**
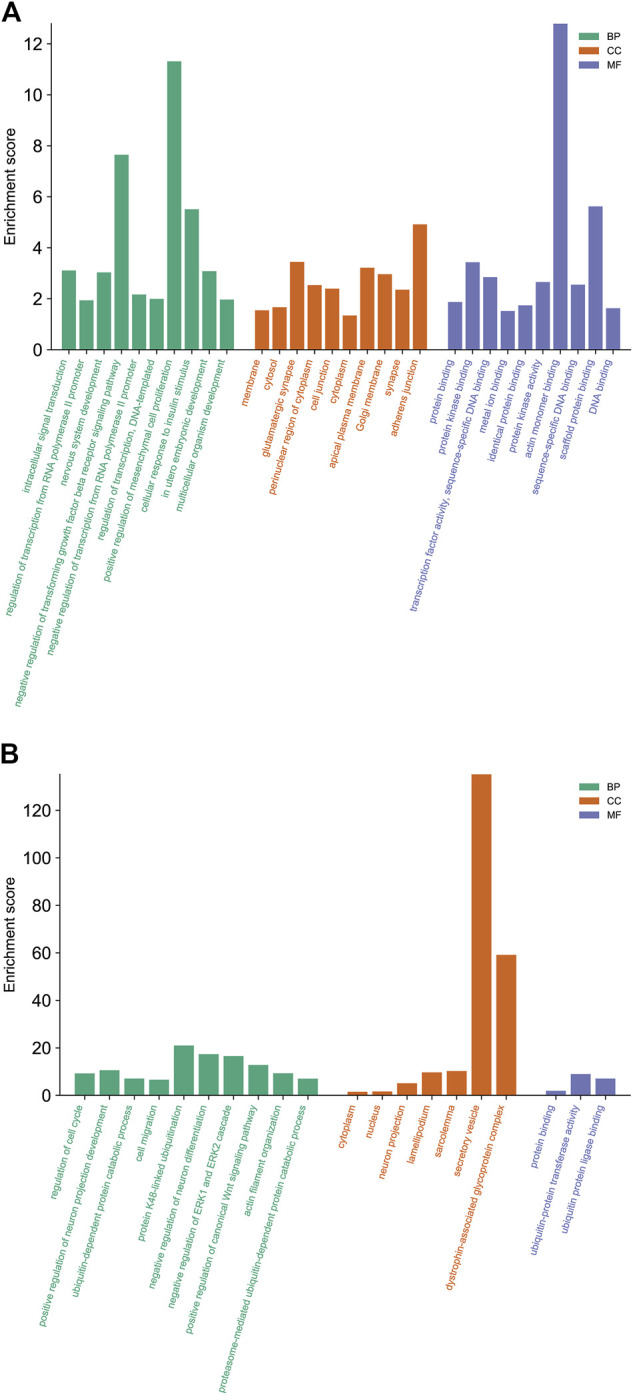
The biological functions analysis of differential expressed miRNAs target genes. The significantly enriched GO biological processes of upregulated miRNAs **(A)** and downregulated miRNAs **(B)**. GO, Gene Ontology; BP, biological process; CC, cellular component; MF, molecular function; KEGG, Kyoto Encyclopedia of Genes and Genomes.

**FIGURE 7:**
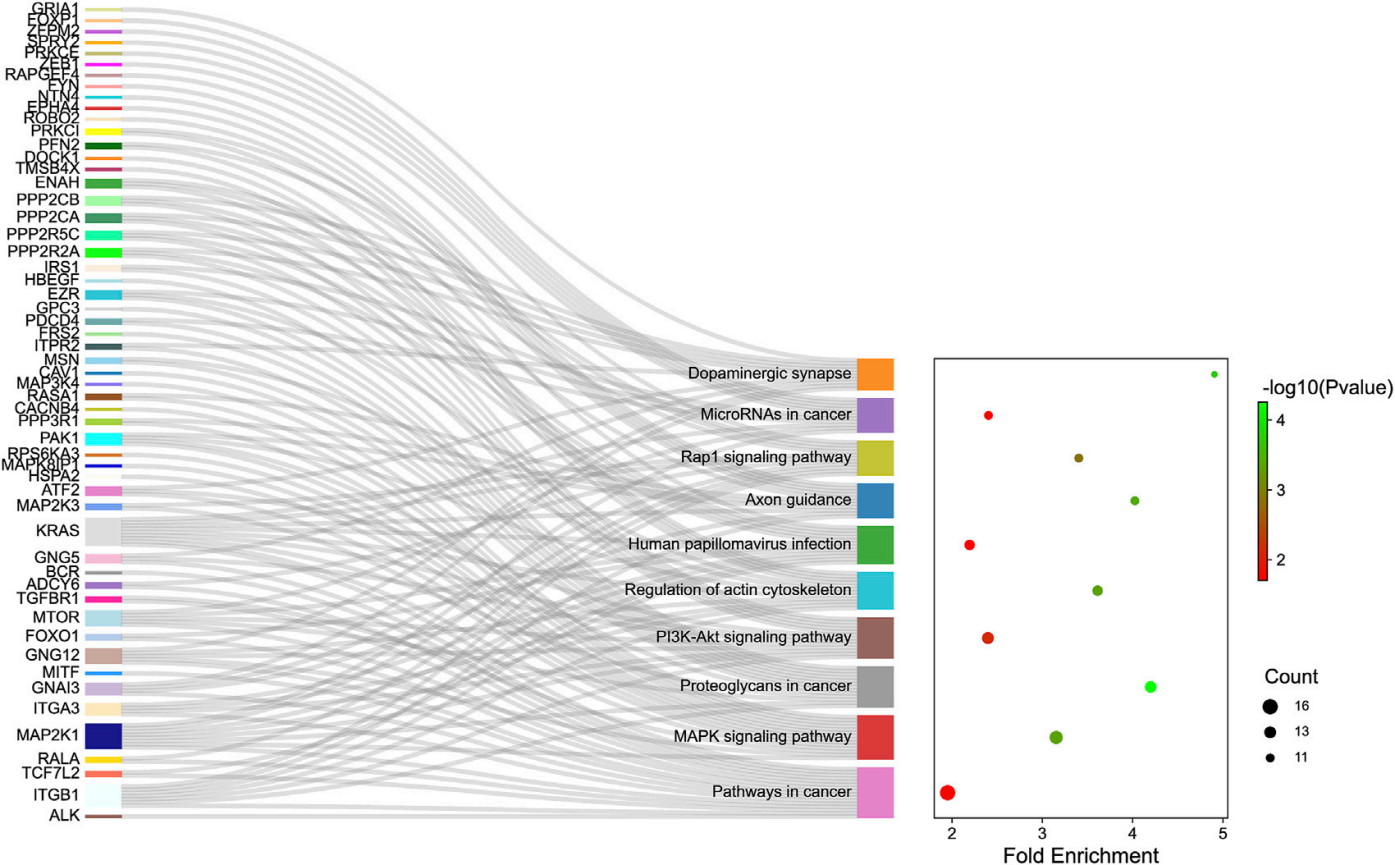
The KEGG enrichment analysis of up-regulated genes. The *y*-axis and *x*-axis depict KEGG-enriched terms and the Gene Ratio, respectively. The size of the dot means the number of genes and the color of the dots represents the *p*-value. The left cluster plot shows a chord dendrogram of clustering the expression spectrum of significantly DE genes. DE, differentially expressed; KEGG, Kyoto Encyclopedia of Genes and Genomes.

**FIGURE 8:**
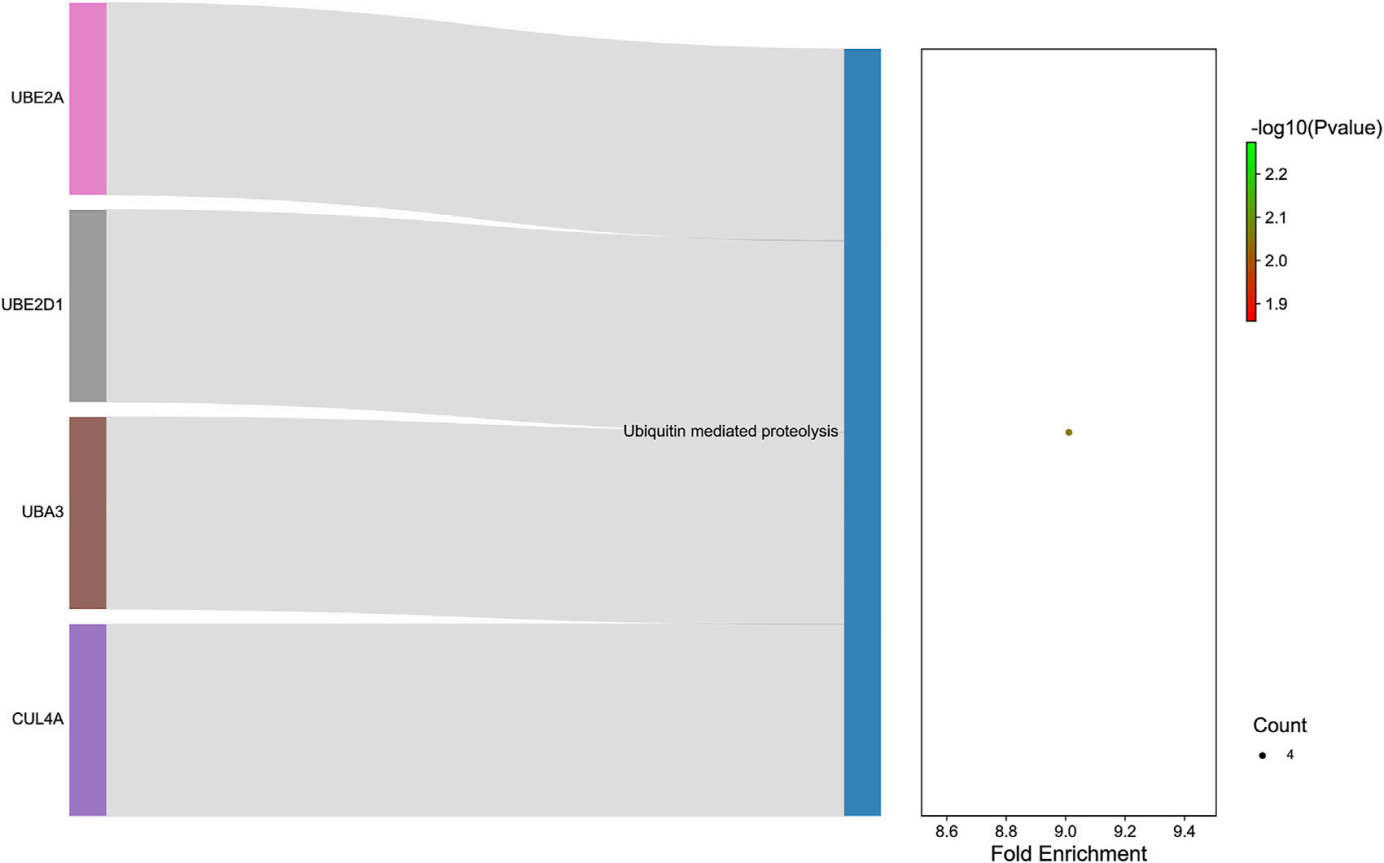
:The KEGG enrichment analysis of down-regulated genes. The *y*-axis and *x*-axis depict KEGG-enriched terms and the Gene Ratio, respectively. The size of the dot means the number of genes and the color of the dots represents the *p*-value. The left cluster plot shows a chord dendrogram of clustering the expression spectrum of significantly DE genes. DE, differentially expressed; KEGG, Kyoto Encyclopedia of Genes and Genomes.

GO analysis was carried out to explain the biological process (BP), cellular components (CC), and molecular functions (MF) of upregulated miRNAs and downregulated miRNAs, respectively. The GO analysis of upregulated miRNAs: most enrichment term of the BP was positive regulation of mesenchymal cell proliferation (GO:0,002,053); the most enriched term of CC was adherens junction (GO:0,005,912); the most enriched term of MF was ubiquitin-protein transferase activity (GO:0,004,842) (Figure 6A and Table 4). The GO analysis of downregulated miRNAs: most enrichment term of the BP was protein K48-linked ubiquitination (GO:0,070,936); the most enriched term of CC was secretory vesicle (GO:0,099,503); the most enriched term of MF was actin monomer binding (GO:0,003,785) (Figure 6B and Table 5). KEGG pathway analysis showed that the upregulated miRNAs were mainly enriched in the MAPK signaling pathway (path: mmu04010) (Figure 7 and Table 6) and the downregulated miRNAs were mainly enriched in the Ubiquitin mediated proteolysis (path: mmu04120) (Figure 8 and Table 7).

**TABLE 4:** GO enrichment analysis of upregulated miRNAs.

ID	Term	subgroup	*p*_value
GO:0035556	intracellular signal transduction	BP	2.70E-05
GO:0006357	regulation of transcription from RNA polymerase II promoter	BP	3.41E-05
GO:0007399	nervous system development	BP	6.20E-05
GO:0030512	negative regulation of transforming growth factor beta receptor signaling pathway	BP	8.20E-05
GO:0000122	negative regulation of transcription from RNA polymerase II promoter	BP	1.37E-04
GO:0006355	regulation of transcription, DNA-templated	BP	1.60E-04
GO:0002053	positive regulation of mesenchymal cell proliferation	BP	1.72E-04
GO:0032869	cellular response to insulin stimulus	BP	2.34E-04
GO:0001701	in utero embryonic development	BP	4.07E-04
GO:0007275	multicellular organism development	BP	4.18E-04
GO:0016020	membrane	CC	4.63E-10
GO:0005829	cytosol	CC	1.11E-07
GO:0098978	glutamatergic synapse	CC	3.27E-07
GO:0048471	perinuclear region of cytoplasm	CC	1.24E-05
GO:0030054	cell junction	CC	1.62E-05
GO:0005737	cytoplasm	CC	2.36E-05
GO:0016324	apical plasma membrane	CC	2.98E-05
GO:0000139	Golgi membrane	CC	3.24E-05
GO:0045202	synapse	CC	4.51E-05
GO:0005912	adherens junction	CC	8.59E-05
GO:0005515	protein binding	MF	4.32E-18
GO:0019901	protein kinase binding	MF	5.23E-08
GO:0003700	transcription factor activity, sequence-specific DNA binding	MF	5.22E-06
GO:0046872	metal ion binding	MF	8.04E-05
GO:0042802	identical protein binding	MF	1.24E-04
GO:0004672	protein kinase activity	MF	2.08E-04
GO:0003785	actin monomer binding	MF	5.72E-04
GO:0043565	sequence-specific DNA binding	MF	7.47E-04
GO:0097110	scaffold protein binding	MF	0.001527
GO:0003677	DNA binding	MF	0.001592

BP, biological process; CC, cellular component; MF, molecular function.

**TABLE 5:** GO enrichment analysis of downregulated miRNAs.

ID	Term	subgroup	*p*_value
GO:0051726	regulation of cell cycle	BP	0.001862
GO:0010976	positive regulation of neuron projection development	BP	0.00594
GO:0006511	ubiquitin-dependent protein catabolic process	BP	0.008601
GO:0016477	cell migration	BP	0.010379
GO:0070936	protein K48-linked ubiquitination	BP	0.012375
GO:0045665	negative regulation of neuron differentiation	BP	0.013565
GO:0070373	negative regulation of ERK1 and ERK2 cascade	BP	0.017501
GO:0090263	positive regulation of canonical Wnt signaling pathway	BP	0.021192
GO:0007015	actin filament organization	BP	0.022047
GO:0043161	proteasome-mediated ubiquitin-dependent protein catabolic process	BP	0.039486
GO:0005737	cytoplasm	CC	0.018553
GO:0005634	nucleus	CC	0.009726
GO:0043005	neuron projection	CC	0.005566
GO:0030027	lamellipodium	CC	0.007694
GO:0042383	sarcolemma	CC	0.033447
GO:0099503	secretory vesicle	CC	0.014373
GO:0016010	dystrophin-associated glycoprotein complex	CC	0.032557
GO:0005515	protein binding	MF	5.59E-04
GO:0004842	ubiquitin-protein transferase activity	MF	0.002083
GO:0031625	ubiquitin protein ligase binding	MF	0.004929

BP, biological process; CC, cellular component; MF, molecular function.

**TABLE 6:** KEGG enrichment analysis of upregulated miRNAs.

**Term**	***p*_value**	**Genes**	**Count**
Pathways in cancer	0.015703202	ALK/ITGB1/TCF7L2/RALA/MAP2K1/ITGA3/GNAI3/MITF/GNG12/FOXO1/MTOR/TGFBR1/ADCY6/BCR/GNG5/KRAS	16
MAPK signaling pathway	4.50E-04	MAP2K3/ATF2/MAP2K1/HSPA2/GNG12/TGFBR1/MAPK8IP1/RPS6KA3/PAK1/PPP3R1/CACNB4/RASA1/KRAS/MAP3K4	14
Proteoglycans in cancer	5.55E-05	ITGB1/MAP2K1/CAV1/MSN/ITPR2/FRS2/MTOR/PAK1/PDCD4/GPC3/KRAS/EZR/HBEGF	13
PI3K-Akt signaling pathway	0.007524932	ITGB1/ATF2/MAP2K1/IRS1/ITGA3/PPP2R2A/PPP2R5C/GNG12/MTOR/PPP2CA/PPP2CB/GNG5/KRAS	13
Regulation of actin cytoskeleton	4.51E-04	ENAH/ITGB1/MAP2K1/PAK1/ITGA3/TMSB4X/MSN/KRAS/GNG12/EZR/DOCK1/PFN2	12
Human papillomavirus infection	0.019858819	ITGB1/PPP2CA/TCF7L2/PPP2CB/PRKCI/MAP2K1/ITGA3/PPP2R2A/KRAS/PPP2R5C/FOXO1/MTOR	12
Axon guidance	3.76E-04	ROBO2/ENAH/ITGB1/EPHA4/PPP3R1/PAK1/RASA1/NTN4/GNAI3/FYN/KRAS	11
Rap1 signaling pathway	0.001376394	ENAH/ITGB1/MAP2K3/PRKCI/MAP2K1/RALA/GNAI3/KRAS/ADCY6/PFN2/RAPGEF4	11
MicroRNAs in cancer	0.01550627	MAP2K1/ZEB1/IRS1/PRKCE/PDCD4/SPRY2/KRAS/ZFPM2/EZR/MTOR/FOXP1	11
Dopaminergic synapse	1.85E-04	PPP2CA/GRIA1/ATF2/PPP2CB/GNG5/GNAI3/ITPR2/PPP2R2A/PPP2R5C/GNG12	10

**TABLE 7:** KEGG enrichment analysis of downregulated miRNAs.

**Term**	***p*_value**	**Genes**	**Count**
Ubiquitin mediated proteolysis	0.008581578	CUL4A/UBA3/UBE2D1/UBE2A	4

### The Expression of miR-96-5p, miR-183-5p, and miR-142a-5p in BV2 Cells With Scratch Injury

**FIGURE 9:**
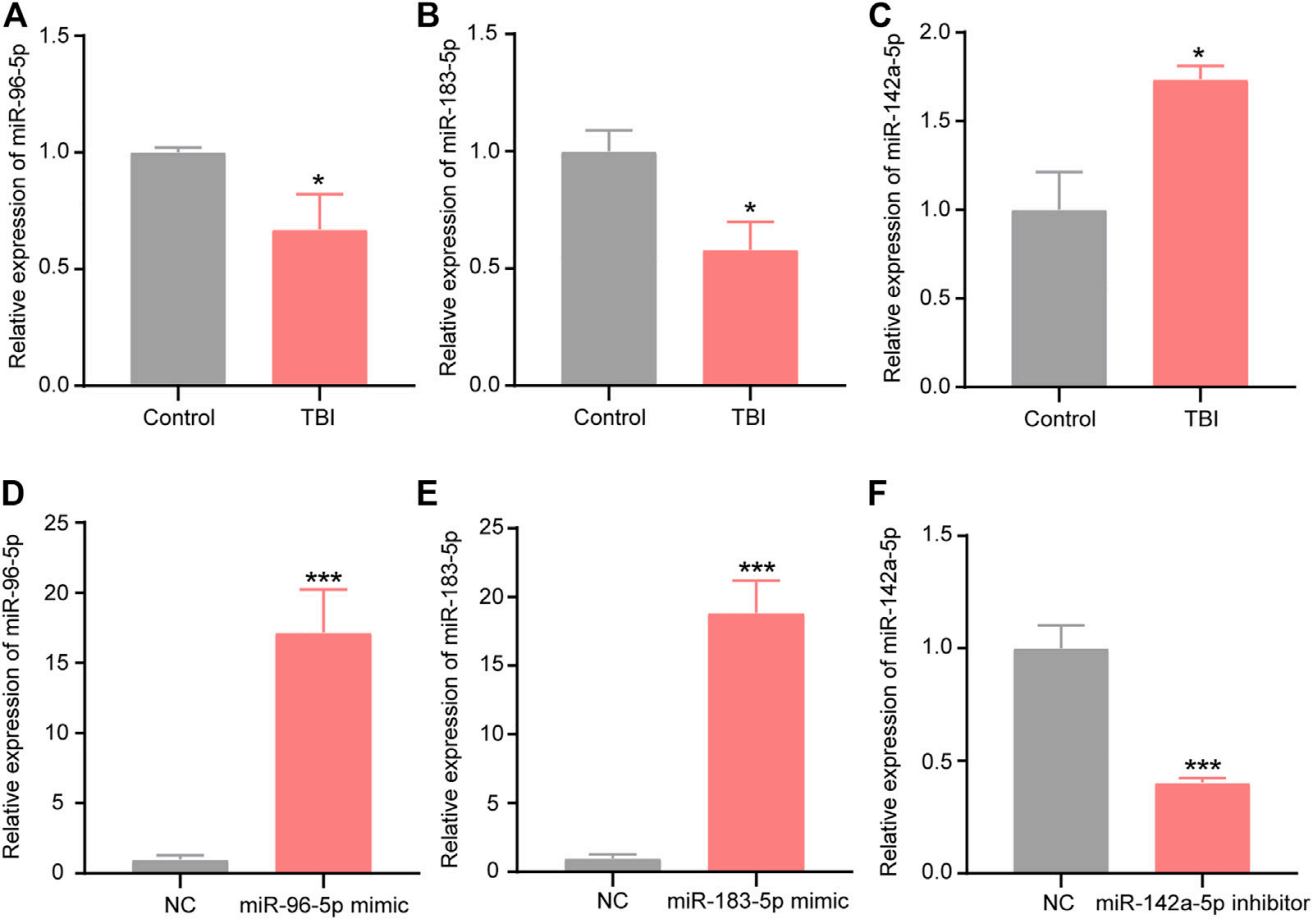
Expression of miR-96-5p **(A)**, miR-183-5p **(B)**, and miR-142a-5p **(C)** in BV2 cells with scratch injury (*p < 0.05, ***p < 0.01 presented TBI compared to control group). qRT-PCR was used to detect the expression of miR-96-5p **(D)**, miR-183-5p **(E)**, and miR-142a-5p **(F)** in the BV2 cells transfected with miR-96-5p mimic, miR-183-5p mimic, and miR-142a-5p inhibitor (*p < 0.05, ***p < 0.01 presented miRNAs compared to NC group). The data are exhibited as the mean ± SEM (*n* = 3).

To initially explore the expression characteristics of miR-96-5p, miR-183-5p, and miR-142a-5p in BV-2 cells with scratch injury, we used the qRT-PCR to detect the levels of miR-96-5p, miR-183-5p, and miR-142a-5p. The expression of miR-96-2p (Figure 9A, *p* < 0.05) and miR-183-5p (Figure 9B, *p* < 0.05) were decreased after scratch injury, but the levels of miR-142a-5p (Figure 9C, *p* < 0.05) was increased after scratch injury. Then we transfected the miR-96-5p mimic, miR-183-5p mimic, and miR-142a-5p inhibitor into the BV-2 cells. To demonstrate the transfection effect of miR-96-5p mimic, miR-183-5p mimic, and miR-142a-5p inhibitor, we used qRT-PCR to detect the expression levels of miR-96-5p, miR-183-5p, and miR-142a-5p in BV-2 cells after transfection. In BV-2 cells, transfection of mimic resulted in an obvious increase in the expression level of miR-96-5p (Figure 9D) and miR-183-5p (Figure 9E). Conversely, the expression level of miR-142a-5p showed a significant decrease following transfection of inhibitor (Figure 9F).

### Overexpression of miR-96-5p Attenuated the Expression of Proinflammatory Factors Induced by Scratch Injury in Microglia

**FIGURE 10:**
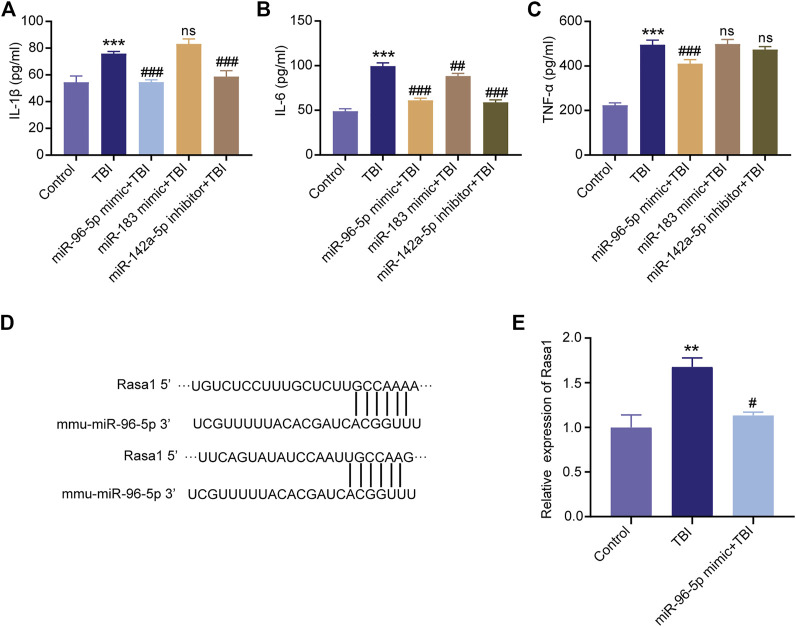
Overexpression of miR-96-5p or miR-183-5p and inhibition of miR-142a-5p suppress the expression of inflammatory cytokines. ELISA was applied to detect the levels of IL-1β **(A)**, IL-6 **(B)**, and TNF-α **(C)** in the BV2 cells culture medium. **(D)** The binding region and seed sequence between miR-96-5p and Rasa1. **(E)** Validation of Rasa1’s levels by qRT-PCR. miRNAs could silence the mRNAs, Rasa1 was significantly upregulated in scratch injury and downregulated in miR-96-5p mimic. The data are exhibited as the mean ± SD (*n* = 3). ***p < 0.01, TBI group vs. control group; ^##^ *p* < 0.01 and ^###^ *p* < 0.001, miRNAs mimic or inhibitor groups vs. TBI group.

Since miR-96-5p, miR-183-5p were significantly up-regulated and miR-142a-5p was down-regulated in the XFZYD group relative to the TBI group, we hypothesized that these three miRNAs played an important role in protective effects after TBI. Interestingly, we found that the expression of miR-96-5p, miR-183-5p were low expressed and miR-142a-5p was overexpressed in BV-2 microglia after the scratch injury (Figures 9A–C). To further explore the functions of the three miRNAs in microglial, we transfected miR-96-5p mimic, miR-183-5p mimic, and miR-142a-5p inhibitor into the BV-2 microglia with scratch injury to further activate microglia. Subsequently, the pro-inflammatory factors IL-1β, IL-6, and TNF-α in cell culture supernatant were detected by ELISA. The results showed that expressions of IL-1β, IL-6, and TNF-α were significantly up-regulated in the microglia with scratch injury (***p < 0.001) but down-regulated when microglia was transfected with miR-96-5p mimic (Figures 10A–C, ^###^ *p* < 0.001). BV-2 microglia transfected with miR-183-5p mimic suppressed the levels of IL-6 (Figure 10B, ^##^ *p* < 0.01) but did not affect changes of IL-1β (Figure 10A) and TNF-α (Figure 10C). BV-2 microglia transfected with miR-142a-5p inhibitor could eliminated the expressions of IL-1β (Figure 10A, ^###^ *p* < 0.001) and IL-6 (Figure 10B, ^###^ *p* < 0.001), but the expression of TNF-α in BV-2 cells was not affected by miR-142a-5p inhibitor (Figure 10C).

### miR-96-5p Target on Rasa1

To validate the mechanism by which miR-96-5p regulates inflammatory responses of microglia, we analyzed the downstream targets of miR-96-5p via the TargetScan (http://www.targetscan.org/) and miRDB (http://www.mirdb.org/miRDB/). The results show that Rasa1 was one of the potential targets of miR-96-5p (Figure 10D). Research demonstrated that miRNAs could recognize target mRNAs and repress their translation via conserved complementary sequence matching. The expression of Rasa1 was further detected by qRT-PCR. We found that the level of Rasa1 was significantly up-regulated in BV-2 cells with scratch injury; while under the effects of miR-96-5p mimic, Rasa1 expression was down-regulated (Figure 10E).

## Discussion

To detect the potential therapeutic approach of XFZYD, we investigated the expression profiles of miRNAs in the hippocampus of TBI and XFZYD treated groups. In the present study, 16 miRNAs were XFZYD-treatment miRNAs. Furthermore, the bioinformatics analysis pointed out that the miRNAs could play roles by regulating cell migration, glutamatergic synapse, protein kinase binding, MAPK signaling pathway, etc. These findings make us deeply understand the XFZYD therapeutic targets and pharmacological mechanisms after TBI.

XFZYD, a traditional Chinese medicine, is recorded in Wang Qing ren’s “Yi Lin Gai Cuo.” Our previous work disclosed the traditional dosage of XFZYD significantly improved spatial learning and memory impairments (Zhu et al., 2018). Evidence-based investigations have manifested that XFZYD can ameliorate neurological recovery post-TBI (Zhou et al., 2017; Zhu et al., 2018). The mNSS of the XFZYD group decreased relative to that of the TBI group on the 3rd (*p* < 0.01), 7th (*p* < 0.05), and 14th days (*p* < 0.01) (Figure 2A). According to the results of the MWM test, The TBI mice exhibited significantly less dwell time in the target quadrant than a sham and XFZYD groups, indicating the retention of spatial and acquired memory was impaired (Figure 2B). A single-factor ANOVA revealed none of the groups’ swimming speeds significantly differed (Figure 2C). The result suggests differences of time in the target quadrant were not due to injury-induced motor impairment. HE staining states that the morphology of the sham group was the round and intact nucleus. In the TBI group, there was severe nuclear concentration, loose staining, and cell death. The pathological degree of the CA1 region in the XFZYD-therapy group was prominently reduced contrasted the TBI group (Figure 2D). Our results are consistent with the previous experiment, indicating the reliability of this study.

Despite animal models being unable to fully simulate the trauma to the human cerebrum, they remain the basis for comprehending the molecular and cellular mechanisms following TBI (Ma et al., 2019). The CCI model is a standard TBI animal model that takes advantage of TBI-0310 to induce damage to the exposed dura (Romine et al., 2014). This model confers duplicatable impairments and mimics numerous features of human trauma such as acute cerebral hemorrhage, blood-brain barrier breakdown, cortical tissue loss, intracranial hypertension, and axonal damage (Osier and Dixon, 2016; Siebold et al., 2018; Lu and Mao, 2019). The CCI model also results in a great many neural functions defects customary in human trauma patients, such as cognitive and motor complications (Adelson et al., 2013; Chen et al., 2014). We established a CCI mouse model as previously reported (Yang et al., 2010). The mNSS and MWM tests indicated that TBI induces neurological deficits in mice (Figures 2A–C). HE staining revealed that TBI leads to brain lesions (Figure 2D), which was quantitatively in good agreement with earlier reports (Xie et al., 2019). It was indicated that the animal model of CCI in our study was reliable.

Traumatic brain injury triggers multitudinous molecular and biochemical alterations during the whole of the central nervous system, including changed transcript expression, disturbed signal communication, affected cell process, and perturbed neurogenesis (Dash et al., 2004; Raghupathi, 2004; Richardson et al., 2007; Redell et al., 2009). Proteomics has been applied to investigate the pathophysiology of TBI. Nonetheless, there could be differences between mRNAs and their related protein products expression levels (Gygi et al., 1999; Redell et al., 2009). To some degree, the distinctions of mRNA and proteins could be ascribed to the roles of miRNAs (Redell et al., 2009). miRNAs, a class of small ncRNAs, control diverse biological action. miRNAs are particularly attractive due to their interactions with their target genes (Liu et al., 2014). Increasing evidence exhibited that miRNAs are engaged in neurological disorders, like Alzheimer’s disease (Gupta et al., 2017), stroke (Mirzaei et al., 2018), and TBI (Xiao X. et al., 2019). Thereby, discerning miRNAs, related targets genes, and their regulatory signaling pathways are crucial in knowing the common biological development of miRNAs and their actions in the disease process (Croce, 2009; Liu et al., 2014). Nowadays, scientists have explored the traditional Chinese Medicine (TCM) pharmacological mechanism and potential therapeutic targets via transcriptomics technologies (Zhang et al., 2017b; Xu, 2017; Li et al., 2018). miRNA-based therapeutics approaches have been assessed at the preclinical and clinical stages. However, trials found single-target strategy could not effectively hinder the development of diseases since additional miRNAs could also affect the target and interfere with the disease’s pathophysiological (Peng et al., 2019). Furthermore, scientists and clinicians regarded that combination therapies for multiple pathological processes might be more practical than single-target treatment in ameliorating neurobehavioral outcomes after TBI (Lagraoui et al., 2017). TCM performs efficient therapies through multiple targets (Li et al., 2020). In the past decades, related findings proved that TCM might affect multiple miRNAs simultaneously (Qian et al., 2013; Momtazi et al., 2016; Peng et al., 2019). Therefore, exploring differentially expressed miRNAs induced by XFZYD in the hippocampus of mice will provide a new direction into the TBI treatment.

RNA-seq supported a platform to analyze a lot of miRNAs simultaneously, comprehensively evaluating potential alterations in expression and generating miRNA expression characteristics for TBI. In this study, 16 differentially expressed miRNAs were found between TBI and XFZYD groups. miR-96-5p, miR-182-5p, miR-183-5p, miR-7015-5p, and miR-296-5p were the five most significant up-regulated miRNAs, while miR-383-5p, miR-142a-5p, miR-33-5p, miR-466d-5p, and miR-466n-5p were the five most significant down-regulated miRNAs (Table 3). Previous references demonstrated that miR-96-5p could regulate spinal cord injury through the NF-κB pathway (Huang et al., 2019) and decreased LPS-induced inflammatory responses (Chen et al., 2020). We identified that miR-96-5p was reduced in TBI and raised in XFZYD treatment. We found that the expression of miR-96-5p was down-regulated in BV-2 cells with scratch injury (Figure 9A). To further explore the functions of the three miRNAs in microglial, we transfected miR-96-5p mimic into the BV-2 microglia with scratch injury to further activate microglia. Subsequently, the pro-inflammatory factors IL-1β, IL-6, and TNF-α in cell culture supernatant were detected by ELISA. The results showed that expressions of IL-1β, IL-6, and TNF-α were significantly up-regulated in the microglia with scratch injury (*p* < 0.001) but down-regulated when microglia was transfected with miR-96-5p mimic (Figures 10A–C, *p* < 0.001). Lin *et al.* (Lin et al., 2017) have shown that miR-183-5p was raised after ischemic post-conditioning. In addition, the enhancement of miR-183-5p relives neuronal deficits after ischemia-reperfusion. Li *et al.* (Zhu et al., 2020) also verified that miR-183-5p expression was decreased in ischemic mice and reduced ischemic injury by negatively regulating PTEN. Wang *et al.* (Wang et al., 2020b) have proved that miR-183-5p decreased after intracerebral hemorrhage (ICH). What’s more, miR-183-5p hinders heme oxygenase-1 to improve neurological damage after ICH. Our study also found that XFZYD treatment could elevate the levels of miR-183-5p. BV-2 microglia transfected with miR-183-5p mimic suppressed the levels of IL-6 (Figure 10B, ^##^ *p* < 0.01) but did not affect changes of IL-1β (Figure 10A) and TNF-α (Figure 10C). miR-142a-5p, one of the miR-142 isoforms, is notably elevated in the context of autoimmune neuroinflammation (Talebi et al., 2017). Evidenced-based results demonstrated miR-142a-5p is associated with immune response. TBI induces brain injury itself and alters the immune response (Sharma et al., 2019). Expression of miR-142-5p was significantly increased in the frontal white matter from multiple sclerosis patients compared with white matter from non-multiple sclerosis controls. Increasing expression of miR-142 isoforms might be involved in the pathogenesis of autoimmune neuroinflammation by influencing T cell differentiation (Talebi et al., 2017). Our study also found miR-142a-5p was upregulated in TBI and downregulated by XFZYD intervention. BV-2 microglia transfected with miR-142a-5p inhibitor could eliminated the expressions of IL-1β (Figure 10A, *p* < 0.001) and IL-6 (Figure 10B, *p* < 0.001), but the expression of TNF-α in BV-2 cells was not affected by miR-142a-5p inhibitor (Figure 10C). However, we have not found any previous research investigating the miR-96-5p, miR-183-5p, and miR-142a-5p in TBI models of animals or humans. Their specific biological functions in TBI deserve to be further investigated. Further studies exploring the relevance of the above miRNAs in TBI will better understand TBI’s biological mechanisms and put insight into novel therapeutic targets for TBI.

Pathway analysis showed that the miRNAs were mainly enriched in the MAPK signaling pathway (path: mmu04010) (Figure 7 and Table 6). The mitogen-activated protein kinase (MAPK) signaling controls extensive biological processes, including growth, differentiation, oxidative stress, and neuroinflammation (Sun and Nan, 2016). Growth, inflammation, and stress response are processes triggered by TBI that are a crucial component of the overall pathophysiology. A large body of evidence suggested that the MAPK signaling pathway regulates inflammation response (Tao et al., 2018), cell apoptosis, and death (Zhang et al., 2020) in TBI. Previous research illustrated that the MAPK signaling pathway is involved in long-term memory (Walz et al., 1999). The recent study also indicated that TCM alleviated the learning and memory in Alzheimer’s disease through the MAPK pathway (Gao et al., 2019). However, the relationship between the MAPK pathway and XFZYD has not been documented. Further studies exploring the relevance of the MAPK in the TBI and XFZYD treatment will better understand the underlying mechanism of XFZYD.

RASA1 is a member of the RAS GTPase Activating Protein (RAS-GAP) family. The well-known oncoprotein RAS can be inactivated by binding to RAS-GAP members. Some studies have shown that mutation or loss of function of RASA1 leads to activation of the RAS-MAPK cascade in malignant tumors (Xiao W. et al., 2019). Dai et al. (Dai et al., 2019) have identified that lncRNA GAS5 served as a competing endogenous RNA (ceRNA) to upregulate Rasa1 via sponging miR-335 in the progression of TBI. Rasa1 was one of the potential targets of miR-96-5p (Figure 10D). We found that the level of Rasa1 was significantly up-regulated in BV-2 cells with scratch injury; while under the effects of miR-96-5p mimic, Rasa1 expression was down-regulated (Figure 10E).

The present study has several limitations. To begin with, the current study has only completed the functional predictions and expression profile; therefore, the next step is determining the roles of the alternative miRNAs in vitro-and-in vivo. Second, clinical samples and large sample sizes will be required to validate the current findings in future studies. More research is needed to explore the particular interactions and sites of binding between mRNAs and miRNAs. Besides, future studies should focus on the link between miR-96-5p and XFZYD treatment.

## Conclusion

Herein, we explored the expression profiles of miRNAs in experimental TBI treated with XFZYD. In comparison to TBI, 16 miRNAs were considerably XFZYD therapy-related. miRNAs could be new therapeutic targets for XFZYD in treating TBI-induced cellular processes. The current study lays the groundwork for future research into the methods through which XFZYD protects against long-term neurological deficits following TBI.

## Data Availability

The datasets presented in this study can be found in online repositories. The names of the repository/repositories and accession number(s) can be found below: National Center for Biotechnology Information (NCBI) BioProject database under accession number GSE198915.
